# Incidence and predictors of left ventricular function change following ST-segment elevation myocardial infarction

**DOI:** 10.3389/fcvm.2023.1079647

**Published:** 2023-03-30

**Authors:** Chuanfen Liu, Meng Guo, Yuxia Cui, Manyan Wu, Hong Chen

**Affiliations:** ^1^ Department of Cardiology, Peking University People’s Hospital, Beijing, China; ^2^ Center for Cardiovascular Translational Research, Peking University People’s Hospital, Beijing, China; ^3^ Beijing Key Laboratory of Early Prediction and Intervention of Acute Myocardial Infarction, Peking University People’s Hospital, Beijing, China

**Keywords:** ST-segment elevation myocardial infarction, primary PCI, echocardiography, left ventricular ejection fraction, left ventricular recovery

## Abstract

**Aim:**

The purpose of the study was to assess the incidence and predictors of left ventricular function change in patients with ST-segment elevation myocardial infarction (STEMI) undergoing primary PCI.

**Methods:**

312 patients with STEMI who received primary percutaneous coronary intervention (PCI) between January 2015 and December 2016 were consecutively enrolled in this study. Multiple logistic regression analysis was used to evaluate independent predictors of left ventricular ejection fraction (LVEF) improvement after long-term follow-up.

**Results:**

We finally analyzed the LVEF change in 186 patients from baseline to follow-up. The mean age was 61.3 ± 12.5 years, with 78.5% being male. The median duration of follow-up after STEMI was 1,021 (389–1,947) days. 54.3% had a decrease in LVEF and 45.7% experienced an improvement in LV function after primary PCI through long-term follow-up. Logistic regression analysis showed lower peak troponin I, non-anterior STEMI, lower baseline LVEF, and no previous myocardial infarction history were independently associated with LVEF improvement.

**Conclusion:**

54.3% of patients with STEMI undergoing primary PCI had a decrease in LVEF during long-term follow-up. LVEF recovery can be predicted by baseline characteristics.

## Introduction

Heart failure (HF) is a major public health problem worldwide. China has experienced an epidemiological transition during the past several decades. The China Hypertension Survey demonstrated that the prevalence of HF is 1.3% in adults ≥35 years (1). The prevalence of HF will continue to increase as the aging of population and the growing incidence of cardiovascular risk factors. With the changes in diseases spectrum in China, the proportion of coronary heart disease and hypertension in the causes of HF is increasing. The China-HF study showed that the proportion of coronary heart disease in patients with HF is 49.6% (2). ST-segment elevation myocardial infarction (STEMI) is an important manifestation of coronary heart disease. Great improvement has been made in the management of STEMI, but HF after STEMI remains a problem. HF early after STEMI is related to adverse prognosis (3). Left ventricular ejection fraction (LVEF) can change dynamically during chronic LV remodeling after STEMI (4). The extent of cardiac chronic remodeling is associated with the development of HF. Primary percutaneous coronary intervention (PCI) is widely used in STEMI nowadays, and LV systolic dysfunction at the remote phase of STEMI remains poorly elucidated. We aim to identify the incidence and predictors of LVEF change several years after PCI in STEMI patients.

## Methods

### Study population

This retrospective study was performed at Peking University People's Hospital in China. Between January 2015 and December 2016, we consecutively enrolled 312 patients with STEMI who received primary PCI. We then screened patients who had both baseline and follow-up LVEF measurements. Exclusion criteria included: without receiving primary PCI; death in the hospital; without baseline and follow-up LVEF values. Diagnosis of STEMI was based on the universal definition of MI (5). The Ethics Review Board of Peking University People's Hospital reviewed and approved this study. All procedures were conducted in accordance with the guidelines of the Helsinki Declaration.

### Data collection

We collected demographic information and clinical characteristics such as medical history, laboratory examinations, and coronary angiography images of the eligible patients. Transthoracic echocardiography was performed during initial hospitalization and at least 3 months after STEMI, and LVEF was estimated by the standard biplane Simpson method. Patients were divided into two groups according to the LVEF change from baseline to follow-up: improvement (ΔLVEF > 0), decline or no recovery (ΔLVEF ≤ 0).

### Statistical analysis

For continuous variables, normally distributed data were reported as the mean ± SD and compared using the Student's *t*-test; non-parametric data are reported as the interquartile range (25%, 75%) and compared using the Mann-Whitney *U*-test. Categorical variables were presented as frequency (%) and compared using the χ^2^ test or Fisher's exact test. Logistic regression analysis was performed to evaluate multivariable predictors of LVEF improvement at follow-up. The variables with *p* < 0.10 in the univariate analysis and clinically significant variables entered in the multivariate analysis. Then the final predictive model was built with the independent predictors, which was developed by assigning weighted points according to the method of risk score establishment proposed in the Framingham Study (6). *p* values < 0.05 was considered statistically significant.

## Results

**Figure 1:**
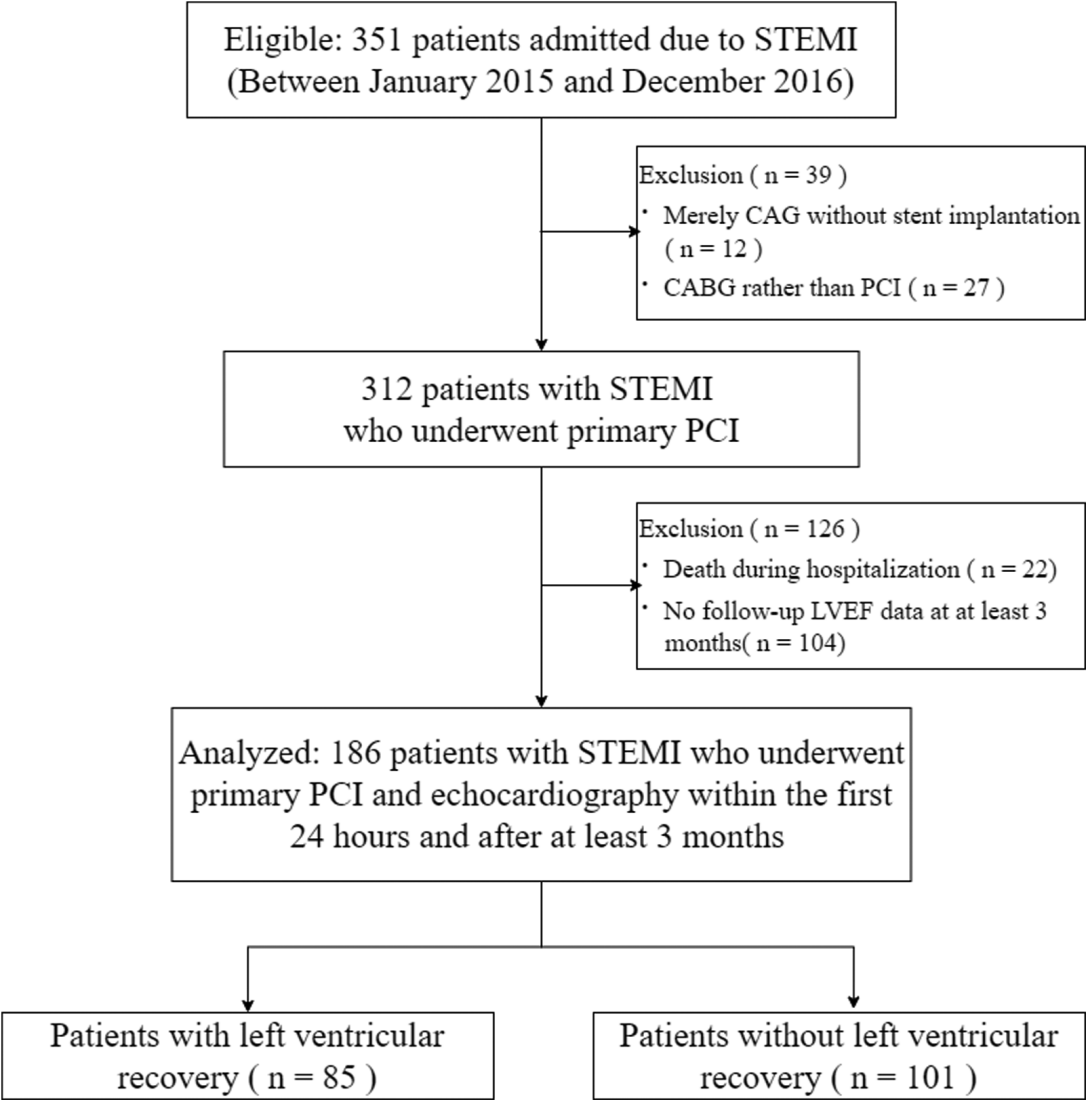
Study flowchart. STEMI, ST-segment elevation myocardial infarction; CAG, coronary angiography; CABG, coronary artery bypass grafting; PCI, percutaneous coronary intervention; LVEF, left ventricular ejection fraction.

Among 312 patients with STEMI who underwent primary PCI were eligible for this study, 126 patients were excluded. Thus, 186 patients were finally analyzed (Figure 1).

### Patient clinical characteristics during index hospitalization for STEMI

Table 1 summarized the baseline clinical characteristics of these patients. The mean age of the patients was 61.3 ± 12.5 years, with 78.5% being male. In terms of cardiovascular risk factors, more than half of the patients had hypertension. 32.3% had type 2 diabetes, and 21.5% had hyperlipemia. The patients whose infarction-related artery was left anterior descending (LAD) accounted for 51.1%. And the patients with left circumflex and right coronary as the culprit vessel were 16.2% and 34.4% respectively. The mean total ischemic time (time from onset-to-balloon) was 8.0 (4.0–24.0) h. And there were 135 (72.6%) patients with TIMI flow grade 3. Most patients received standard medications at discharge. 126 patients were excluded because of death during initial hospitalization (22) and without follow-up LVEF values (104). We compared patients with and without follow-up LVEF values in Supplementary Table S1.

**Table 1:** Baseline clinical characteristics.

Variable	Total (*n* = 186)
Demographics	
Age (year)	61.3 ± 12.5
Male	146 (78.5%)
Body mass index (kg/m^2^)	25.5 ± 3.5
Cardiovascular risk factors	
Hypertension	105 (56.5%)
Diabetes	60 (32.3%)
Hyperlipidemia	40 (21.5%)
Cerebrovascular disease	26 (14.0%)
**Previous myocardial infarction**	20 (10.8%)
**Previous heart failure**	14 (7.5%)
**Previous PCI**	18 (9.7%)
**Previous CABG**	5 (2.7%)
**Heart rate**	76.0 (66.3–85.0)
Hemodynamic parameters	
Systolic blood pressure (mmHg)	118.2 ± 20.9
Diastolic blood pressure (mmHg)	70.3 ± 14.3
Cardiac function (Killip class)	
I	143 (76.9%)
II	30 (16.1%)
III	7 (3.8%)
IV	5 (2.7%)
Culprit vessel	
Left anterior descending	95 (51.1%)
Left circumflex	30 (16.2%)
Right coronary artery	64 (34.4%)
**Multivessel disease**	136 (73.1%)
**Symptom onset-to-balloon time (h)**	8.0 (4.0–24.0)
TIMI flow grade	
3	135 (72.6%)
2	2 (1.1%)
1	1 (0.5%)
0	4 (2.2%)
Laboratory profiles	
Peak troponin I (ng/ml)	18.32 (6.39–72.83)
Peak CK-MB (ng/ml)	73.3 (19.3–236.8)
Fasting blood-glucose (mmol/L)	6.5 (5.3–8.6)
Low-density lipoprotein cholesterol (mmol/L)	2.98 ± 0.88
Estimated Glomerular filtration rate (ml/min*1.73 m^2^)	90.5 (76.7–99.2)
Discharge medication	
Aspirin	182 (97.8%)
P2Y_12_ inhibitor	178 (95.7%)
Statins	183 (98.4%)
Beta-blocker	161 (86.6%)
ACEI/ARB	119 (64.0%)
Diuretic	35 (18.8%)
Nitrates	102 (54.8%)

PCI, percutaneous coronary intervention; CABG, coronary artery bypass graft; TIMI, thrombolysis in myocardial infarction; ACEI, angiotensin converting enzyme inhibitor; ARB, angiotensin receptor blocker.

### Patient echocardiographic characteristics from baseline to follow-up

After a median follow-up of 1,021 (389–1,947) days, although 85 (45.7%) patients showed left ventricular function recovery (i.e., ΔLVEF > 0), there were no significant differences in LV function between baseline and follow-up echocardiography. However, there are more patients with LVEF < 50% at follow-up (*p* < 0.001). Left atrial diameter was greater at follow-up (*p* < 0.001). Another important echocardiographic measurement, the left ventricular end-diastolic diameter, also increased from baseline (*p* = 0.014) (Table 2). Left ventricular wall thickness including interventricular septum and left ventricular posterior wall became thinner (*p* < 0.001).

**Table 2:** Baseline and follow-up echocardiographic parameters.

Parameter	Baseline	Follow-up	*p* value
Left atrial diameter (cm)	3.7 (3.4–3.9)	3.9 (3.5–4.2)	<0.001
Left ventricular end- diastolic diameter (cm)	5.1 (4.8–5.4)	5.2 (4.8–5.6)	0.014
Interventricular septum thickness (cm)	0.98 (0.87–1.10)	0.91 (0.80–1.00)	<0.001
Left ventricular posterior wall thickness (cm)	0.94 (0.88–1.00)	0.89 (0.78–0.98)	<0.001
LVEF (%)	63.4 (56.0–68.0)	63.7 (56.0–68.7)	0.919
LVEF <50%	18 (9.7%)	27 (14.5%)	<0.001
E/A	0.85 (0.68–1.20)	0.76 (0.60–1.10)	0.009
Regional wall motion abnormality	124 (66.7%)	104 (55.9%)	<0.001
Ventricular aneurysm	13 (7.0%)	10 (5.4%)	<0.001

LVEF, left ventricular ejection fraction; E, early diastolic mitral inflow velocity; A, late diastolic mitral inflow velocity.

### Predictors of left ventricular ejection fraction change

85 (45.7%) of all patients showed left ventricular function recovery. In patients with left ventricular function recovery, both the peak troponin I [13.75 (4.93–39.81) vs. 36.30 (8.37–80.0), *p* = 0.010] and peak CK-MB [41.1 (14.3–175.2) vs. 111.5 (28.9–294.0), *p* = 0.008] were significantly lower (Table 3). The symptom onset-to-balloon time was also shorter in these patients [5.33 (3.75–15.50) vs. 11.93 (5.00–24.00), *p *= 0.002].

**Table 3:** Baseline clinical characteristics according to improvement in left ventricular ejection fraction (LVEF).

Variable	Change in LVEF (%)		*p* value
	>0 (*n* = 85)	≤0 (*n* = 101)	
Age (year)	62.5 ± 13.4	60.4 ± 11.6	0.275
Male	68 (80.0%)	78 (77.2%)	0.647
Body mass index (kg/m^2^)	25.4 ± 4.1	25.6 ± 2.9	0.679
Hypertension	44 (51.8%)	61 (60.4%)	0.237
Diabetes	31 (36.5%)	29 (28.7%)	0.260
Hyperlipidemia	21 (24.7%)	19 (18.8%)	0.330
Cerebrovascular disease	12 (14.1%)	14 (13.9%)	0.960
Previous myocardial infarction	8 (9.4%)	12 (11.9%)	0.588
Previous heart failure	7 (8.2%)	7 (6.9%)	0.737
Previous PCI	8 (9.4%)	10 (9.9%)	0.910
Previous CABG	3 (3.5%)	2 (2.0%)	0.834
Symptom onset-to-balloon time (h)	5.33 (3.75–15.50)	11.93 (5.00–24.00)	0.002
Multivessel disease	63 (74.1%)	73 (72.3%)	0.531
LAD as the culprit vessel	38 (44.7%)	59 (58.4%)	0.093
Not TIMI flow grade 3	2 (2.4%)	5 (5.0%)	0.806
Peak troponin I (ng/ml)	13.75 (4.93–39.81)	36.30 (8.37–80.0)	0.010
Peak CK-MB (ng/ml)	41.1 (14.3–175.2)	111.5 (28.9–294.0)	0.008
Aspirin	83 (97.6%)	99 (98.0%)	0.672
P2Y12 inhibitor	80 (94.1%)	98 (97.0%)	0.803
Statins	83 (97.6%)	100 (99.0%)	0.896
Beta-blocker	74 (87.1%)	87 (86.1%)	0.693
ACEI/ARB	51 (60.0%)	68 (67.3%)	0.350
Diuretic	18 (21.2%)	17 (16.8%)	0.446
Nitrates	51 (60.0%)	51 (50.5%)	0.137

PCI, percutaneous coronary intervention; CABG, coronary artery bypass graft; LAD, left anterior descending; TIMI, thrombolysis in myocardial infarction; ACEI, angiotensin converting enzyme inhibitor; ARB, angiotensin receptor blocker.

In univariate analysis, the symptom onset-to-balloon time, the peak troponin I, the peak CK-MB, and baseline LVEF were associated with left ventricular function recovery at follow-up. The variables entered into the multivariate analysis were age, sex, hypertension, diabetes, hyperlipidemia, previous myocardial infarction, symptom onset-to-balloon time, LAD as the culprit vessel, peak troponin I, peak CK-MB, and LVEF at baseline. The multivariate analysis demonstrated that previous history of myocardial infarction (OR: 0.196, 95% CI: 0.039–0.988, *p* = 0.048), LAD as the culprit vessel (OR: 0.212, 95% CI: 0.078–0.578, *p* = 0.002), peak troponin I (OR: 0.978, 95% CI: 0.963–0.992, *p* = 0.002) and LVEF at baseline (OR: 0.881, 95% CI: 0.821–0.947, *p* = 0.001) were independent predictors of LV function recovery (Table 4).

**Table 4:** Logistic regression analysis for the left ventricular recovery.

Univariable	Odds ratio	95% CI	*p* value
Age	1.013	0.990–1.037	0.268
Male	1.179	0.582–2.390	0.647
Body mass index (kg/m^2^)	0.980	0.892–1.077	0.677
Hypertension	0.704	0.393–1.261	0.238
Diabetes	1.425	0.769–2.642	0.260
Hyperlipidemia	1.416	0.702–2.856	0.331
Previous myocardial infarction	0.771	0.299–1.983	0.589
Symptom onset-to-balloon time	0.995	0.990–0.999	0.028
Not TIMI flow grade 3	0.582	0.109–3.107	0.526
Multivessel disease	1.266	0.605–2.647	0.531
LAD as the culprit vessel	0.595	0.323–1.094	0.094
Peak troponin I	0.987	0.978–0.996	0.004
Peak CK-MB	0.996	0.994–0.999	0.007
LVEF at baseline	0.934	0.902–0.967	<0.001
**Multivariable**	**Odds ratio**	**95% CI**	*p* value
Previous myocardial infarction	0.196	0.039–0.988	0.048
LAD as the culprit vessel	0.212	0.078–0.578	0.002
Peak troponin I	0.978	0.963–0.992	0.002
LVEF at baseline	0.881	0.821–0.947	0.001

TIMI, thrombolysis in myocardial infarction; LAD, left anterior descending; LVEF, left ventricular ejection fraction.

### The predictive model for left ventricular function recovery

**Figure 2:**
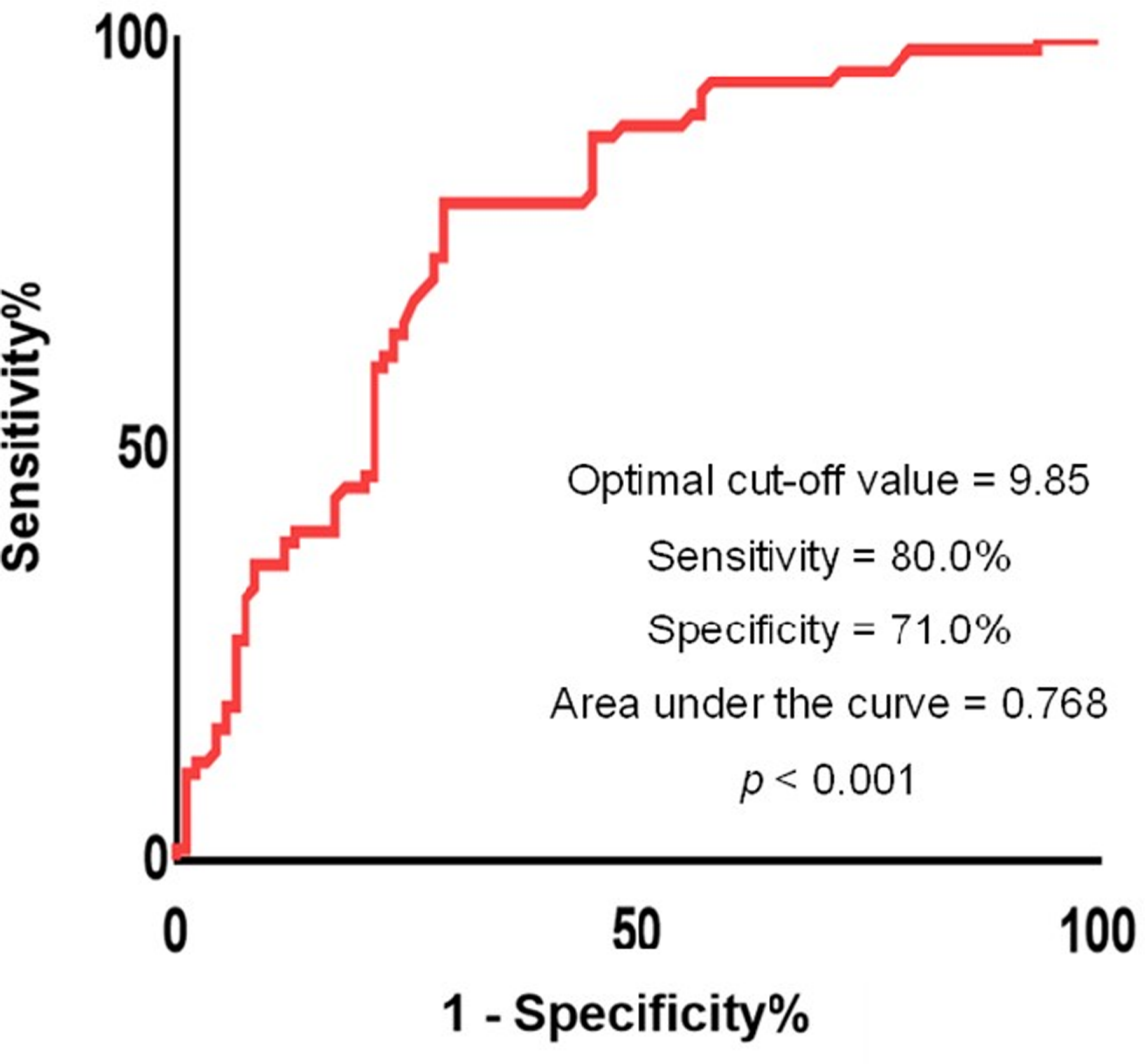
Receiver operating characteristics curves of the proposed predictive model. The optimal threshold of the model for predicting left ventricular recovery was −0.57 with sensitivity 0.800, specificity 0.710, and area under the curve 0.768, respectively (*p* < 0.001).

The points were assigned based on regression coefficients in the multivariate logistic regression model and we established a final predictive model as: 1.628 × previous myocardial infarction + 1.551 × LAD as the culprit vessel + 0.023 × peak troponin I + 0.126 × LVEF at baseline. Using ROC curve analysis, the optimal cut-off value of the predictive model in predicting left ventricular function recovery was 9.85 (sensitivity 0.800, specificity 0.710, positive predictive value 0.690, negative predictive value 0.815, AUC 0.768, 95% CI: 0.697–0.840, *p* < 0.001) (Figure 2).

There were 87 patients with a predictive model score less than 9.85. Compared to the patients with a predictive model score more than 9.85, patients with a predictive model score less than 9.85 had shorter total ischemic time (*p* = 0.005), and lower peak CK-MB (*p* < 0.001). No significant difference was found in age, sex, cardiovascular risk factors, and most medications at discharge between the two groups (Table 5).

**Table 5:** Clinical characteristics of patients according to the optimal cut-off value for the predictive model.

Variable	The score ≤ 9.85	The score > 9.85	*p* value
	(*n* = 87)	(*n* = 81)	
Age (year)	61.7 ± 12.3	59.1 ± 11.6	0.177
Male	69 (79.3%)	66 (81.5%)	0.723
Body mass index (kg/m^2^)	25.9 ± 4.1	25.4 ± 2.5	0.391
Hypertension	52 (59.8%)	43 (53.1%)	0.383
Diabetes	34 (39.1%)	21 (25.9%)	0.069
Hyperlipidemia	16 (18.4%)	21 (25.9%)	0.239
Cerebrovascular disease	11 (12.6%)	11 (13.6%)	0.857
Previous heart failure	6 (6.9%)	7 (8.6%)	0.672
Previous PCI	6 (6.9%)	10 (12.3%)	0.229
Previous CABG	2 (2.3%)	2 (2.5%)	0.932
Symptom onset-to-balloon time (h)	5.5 (3.9–16.0)	8.0 (5.0–48.0)	0.005
Multivessel disease	73 (83.9%)	61 (75.3%)	0.166
Not TIMI flow grade 3	2 (2.3%)	4 (4.9%)	0.523
Peak CK-MB (ng/ml)	39.4 (11.6–135.6)	198.4 (47.3–296.9)	<0.001
Aspirin	87 (100.0%)	79 (97.5%)	0.231
P2Y12 inhibitor	82 (94.2%)	80 (98.8%)	0.247
Statins	47 (54.0%)	60 (74.1%)	0.007
Beta-blocker	74 (85.1%)	73 (90.1%)	0.321
ACEI/ARB	86 (98.9%)	80 (98.8%)	0.959
Diuretic	18 (20.7%)	11 (13.6%)	0.237
Nitrates	49 (56.3%)	42 (51.9%)	0.620

PCI, percutaneous coronary intervention; CABG, coronary artery bypass graft; TIMI, thrombolysis in myocardial infarction; ACEI, angiotensin converting enzyme inhibitor; ARB, angiotensin receptor blocker.

## Discussion

Our study showed in patients with STEMI, 54.3% had a decrease in LVEF and 45.7% experienced an improvement in LVEF after primary PCI through a nearly 4-year follow-up. Second, lower peak troponin I, non-anterior STEMI, lower baseline LVEF, and no previous myocardial infarction history were independently associated with LVEF improvement from baseline to long-term follow-up.

Rapid progress in primary coronary revascularization has reduced mortality in patients with STEMI, while the increased risk of HF after STEMI has become an emerging clinical problem. The change of LVEF after STEMI is a dynamic process. Myocardium stunning following acute coronary artery occlusions affects the recovery of LV function in the early stage of STEMI (7). However, LV remodeling is related to long-term LVEF recovery (8, 9). In our study, we found that after long-term follow-up, 54.3% of patients with STEMI had a decrease in LVEF, suggesting the effect of myocardial infarction on LV function is a long-term process.

A lower peak troponin I independently correlated with LVEF improvement in the present study, consistent with prior studies (10–15). Overwhelming evidences suggest that infarct size, which was quantified directly by Cardiac Magnetic Resonance Images or Single-Photon Emission Computed Tomography, can predict remodeling of the left ventricle (16, 17). The predictive value of peak troponin I is attributed to its ability to estimate infarct size, as previous studies proved peak troponin I correlated with infarct size (18, 19). Our study showed that non-anterior STEMI was an independent predictor of LV function recovery. A study found that an anterior MI location was associated with adverse remodeling (10). Another study also demonstrated that the worse LV function in anterior STEMI patients is due to its larger MI size (20). However, there is also some supporting evidence to suggest that those patients with the culprit vessel as the LAD were more likely to benefit from reperfusion therapy (21, 22). A lower baseline LVEF was associated with LVEF improvement in our study. The reason for this inverse relationship between baseline LVEF and LVEF improvement remains uncertain. This result may be explained by the greater recovery in LVEF after primary PCI in those patients with more ischemic damage (23). Those patients may have a greater potential for functional recuperation (10). At the same time, quite a few studies suggested the opposite results, that is, patients with a higher baseline LVEF were more likely with left ventricular recovery (15, 24). Studies that dynamically observe LVEF changes after primary PCI in STEMI patients may need further exploration.

In the present study, symptom onset-to-balloon time was not an independent predictor of LVEF improvement. Some studies also reported no correlation between time to reperfusion and LVEF improvement (25, 26). In contrast, some studies showed that the time to reperfusion is important for the recovery of left ventricular function (26). It is possible that total ischemic time is related to other measurements such as peak troponin I. It is also likely that early reperfusion is related to early LV function recovery, but in our study, we focused on the remote phase of STEMI.

## Limitation

Several limitations should be noted in our study. First, in this single-center and retrospective study, the sample size is not big enough. Second, left ventricular function measures may have variability. Third, we didn't have time-scheduled LVEF measurements due to the observational design of the study. Finally, the prognostic impact of LVEF change on outcomes in patients with STEMI undergoing primary PCI was not assessed.

## Conclusion

More than half of patients with STEMI had a decrease in LVEF during a long-term follow-up. Lower peak troponin I, non-anterior STEMI, lower baseline LVEF, and no previous myocardial infarction history were independent predictors of LVEF improvement from baseline to follow-up.

## Data Availability

The raw data supporting the conclusions of this article will be made available by the authors, without undue reservation.
