# Supplementary material for: Incidence and predictors of left ventricular function change following ST-segment elevation myocardial infarction
Source: Front Cardiovasc Med. 2023 Mar 30;10:1079647. doi: 10.3389/fcvm.2023.1079647 (PMC10098331; doi:10.3389/fcvm.2023.1079647)
Supplement: Supplementary file 1 [file Table1.docx]

Supplementary Table 1. Patients with and without follow-up LVEF values

| Variable | Patients with follow-up LVEF values  (n = 186) | Patients without follow-up LVEF values  (n = 104) | | P value |
| --- | --- | --- | --- | --- |
| Demographics |  | |  |  |
| Age (y) | 61.3 ± 12.5 | | 65.5 ± 13.7 | 0.126 |
| Male | 146 (78.5%) | | 79 (76.0%) | 0.701 |
| Body mass index (kg/m^2^) | 25.5 ± 3.5 | | 24.9 ± 3.8 | 0.300 |
| Cardiovascular risk factors |  | |  |  |
| Hypertension | 105 (56.5%) | | 65 (62.5%) | 0.279 |
| Diabetes | 60 (32.3%) | | 35 (33.6%) | 0.820 |
| Hyperlipidemia | 40 (21.5%) | | 28 (26.9%) | 0.213 |
| Cerebrovascular disease | 26(14.0%) | | 18 (17.3%) | 0.460 |
| Previous myocardial infarction | 20 (10.8%) | | 13 (12.5%) | 0.737 |
| Previous heart failure | 14 (7.5%) | | 17 (16.3%) | 0.028 |
| Previous PCI | 18 (9.7%) | | 6 | 0.276 |
| Previous CABG | 5(2.7%) | | 3 | 0.928 |
| Heart rate | 76.0 (66.3 - 85.0) | | 76.0 (68.8 - 89.2) | 0.230 |
| Hemodynamic parameters |  | |  |  |
| Systolic blood pressure (mmHg) | 118.2 ± 20.9 | | 120.1 ± 21.2 | 0.392 |
| Diastolic blood pressure (mmHg) | 70.3 ± 14.3 | | 71.5 ± 13.6 | 0.822 |
| Cardiac function (Killip class) |  | |  | < 0.001 |
| Ⅰ | 143 (76.9%) | | 61 (58.6%) |  |
| Ⅱ | 30 (16.1%) | | 23 (22.1%) |  |
| Ⅲ | 7 (3.8%) | | 8 (7.7%) |  |
| Ⅳ | 5 (2.7%) | | 12 (11.5%) |  |
| Culprit vessel |  | |  | 0.046 |
| Left anterior descending | 95 (51.1%) | | 67 (64.4%) |  |
| Left circumflex | 30 (16.2%) | | 8 (7.7%) |  |
| Right coronary artery | 64 (34.4%) | | 29 (27.9%) |  |
| Multivessel disease | 136 (73.1%) | | 63 (60.6%) | 0.417 |
| Symptom onset-to-balloon time (h) | 8.0 (4.0 - 24.0) | | 9.5 (5.0 - 39.0) | 0.261 |
| TIMI flow grade |  | |  | 0.349 |
| 3 | 135 (72.6%) | | 97 (93.3%) |  |
| 2 | 2 (1.1%) | | 5 (4.8%) |  |
| 1 | 1 (0.5%) | | 0 (0.0%) |  |
| 0 | 4 (2.2%) | | 2 (1.9%) |  |
| Laboratory profiles |  | |  |  |
| Peak troponin I (ng/ml) | 18.32 (6.39 - 72.83) | | 17.98 (3.39 - 59.02) | 0.149 |
| Peak CK-MB (ng/ml) | 73.3 (19.3 - 236.8) | | 59.6 (8.5 - 186.8) | 0.153 |
| Fasting blood-glucose (mmol/L) | 6.5 (5.3 - 8.6) | | 6.3 (5.2 - 9.5) | 0.733 |
| ﻿Low-density lipoprotein cholesterol (mmol/L) | 2.98 ± 0.88 | | 2.78 ± 0.91 | 0.208 |
| ﻿estimated Glomerular filtration rate (ml/min*1.73m^2^) | 90.5 (76.7 - 99.2) | | 91.3 (73.7 - 96.8) | 0.151 |
| Discharge medication |  | |  |  |
| Aspirin | 182 (97.8%) | | 101 (97.1%) | 0.736 |
| P2Y_12_ inhibitor | 178 (95.7%) | | 98 (94.2%) | 0.733 |
| Statins | 183 (98.4%) | | 100 (96.2%) | 0.414 |
| Beta-blocker | 161 (86.6%) | | 81 (77.9%) | 0.079 |
| ACEI/ARB | 119 (64.0%) | | 63 (60.1%) | 0.599 |
| diuretic | 35 (18.8%) | | 22 (21.2%) | 0.431 |
| nitrates | 102 (54.8%) | | 58 (55.8%) | 0.924 |
